# Enhancing *in planta* gene targeting efficiencies in Arabidopsis using temperature‐tolerant CRISPR/*Lb*Cas12a

**DOI:** 10.1111/pbi.13426

**Published:** 2020-08-18

**Authors:** Laura Merker, Patrick Schindele, Teng‐Kuei Huang, Felix Wolter, Holger Puchta

**Affiliations:** ^1^ Botanical Institute Karlsruhe Institute of Technology Karlsruhe Germany

**Keywords:** CRISPR, Cas, Cas12, Cpf1, temperature‐tolerant, homologous recombination, double‐strand break repair

The application of the CRISPR/Cas‐system paved the way for the era of genome engineering and quickly became the standardly applied tool for targeted non‐homologous end joining (NHEJ) based mutagenesis. Despite various efforts, homologous recombination (HR) based gene targeting (GT) still requires further improvement regarding efficiency for general applicability in plants (Huang and Puchta, 2019). We developed the *in planta* GT (ipGT) method aimed to establish a technology for crops with meagre transformation efficiencies (Fauser *et al*., 2012). Here, the targeting vector including the nuclease is integrated into the genome and excised at the same time as the target site is activated by double‐strand break (DSB) induction (Figure 1a). We successfully adopted Cas9 for this application (Schiml *et al*., 2014) and improved GT efficiencies by replacing Cas9 from *Streptococcus pyogenes* (*Sp*Cas9) with Cas9 from *Staphylococcus aureus* (*Sa*Cas9), which is more efficient in DSB induction in Arabidopsis (Steinert *et al*., 2015). Egg‐cell specific expression and screening for the most efficient transgenic lines were further key points for GT improvement (Miki *et al*., 2018; Wolter *et al*., 2018). Most recently, we tested the CRISPR/*Lb*Cas12a‐system for ipGT and could demonstrate a further increase in GT efficiencies despite lower InDel rates induced by *Lb*Cas12a compared with *Sa*Cas9 at the *ALS* target locus (Wolter and Puchta, 2019). We speculated that the higher GT efficiency is caused by Cas12a‐mediated cleavage on the PAM distal site, leaving the seed sequence unaffected by mutagenesis. Thus, further cleavage of NHEJ repaired junctions might be much more frequent with Cas12a than with Cas9, enhancing the probability for HR to take over DSB repair. This is also in line with very recently published results on enhancing GT efficiency using *Lb*Cas12a in rice and tomato (Li *et al*., 2019; van Vu *et al*., 2020). Recently, by introducing the single amino acid substitution D156R, we were able to obtain a *Lb*Cas12a nuclease variant with improved, temperature‐tolerant cutting efficiency (tt*Lb*Cas12a) for plant gene editing, outperforming *Lb*Cas12a at 22°C and 28°C in mutation induction (Schindele and Puchta, 2019). Based on those findings, we were interested to test whether replacing *Lb*Cas12a by tt*Lb*Cas12a could further improve GT.

**Figure 1:**
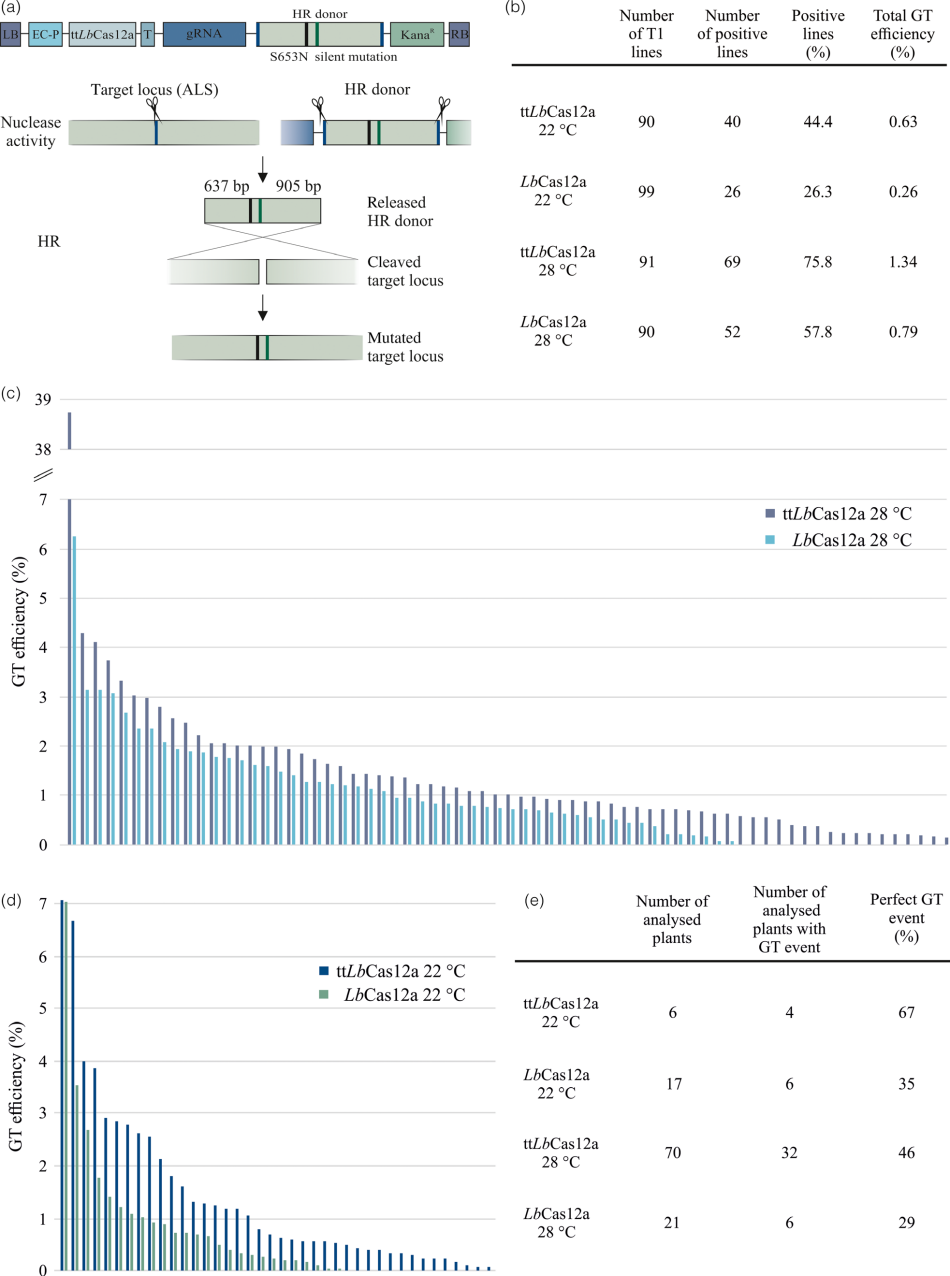
GT efficiencies and molecular analysis. (a) Schematic representation of the ipGT assay. The T‐DNA construct consists of an egg cell‐specific Cas12a expression cassette, followed by a guide RNA expression cassette, the HR donor sequence and a Kanamycin resistance. The HR donor harbours the S653N mutation conferring Imazapyr resistance and a silent mutation preventing cleavage. Homologous regions of 637 bp and 905 bp, both including one mismatch each, are present upstream and downstream of the target DSB on the HR donor. After inducing three DSB, the released HR donor can be used as a template for HR‐mediated DSB repair. (b) Quantitative outcome of the four GT approaches. The amount of analysed independent T1 lines are shown in the first column and the number and percentage of positive lines generating heritable GT events as well as the total GT efficiency in the following. The total GT efficiency describes the average of the GT rates of each single line, including lines without resistant plants. Each GT efficiency was calculated by dividing the number of resistant plants by the total number of T2 seeds. (c) Distributions of GT efficiencies of single lines for tt*Lb*Cas12a and *Lb*Cas12a at 28°C. The positive single lines of the two approaches are arranged from highest to lowest efficiencies, whereby each bar demonstrates the GT efficiency of a single line. GT events were detected in 68 out of 97 lines for tt*Lb*Cas12a and for *Lb*Cas12a in 52 out of 90 analysed lines. (d) Distributions of GT efficiencies of single lines for tt*Lb*Cas12a and *Lb*Cas12a at 22°C. The representation is equivalent to d. GT events were detected in 40 out of 90 analysed lines for tt*Lb*Cas12a and for *Lb*Cas12a in 26 out of 99 analysed lines. (e) Molecular analysis of GT events. The numbers of analysed Imazapyr resistant plants are shown in the first column, followed by the number and percentage of plants, in which a perfect GT event was detected.

Therefore, we performed ipGT experiments in Arabidopsis testing *Lb*Cas12a and tt*Lb*Cas12a at 22°C as well as at 28°C in parallel for a direct comparison. As described before, the acetolactate synthase gene (*ALS*) was selected as target and both nucleases were expressed by an egg‐cell specific promoter to assure efficient germline transmission (Wolter *et al*., 2018). For GT induction, a ribozyme‐flanked crRNA cassette was used with a spacer targeting *ALS*, a respective GT donor molecule and a Kanamycin expression cassette for selection. The donor molecule contains the modification (S653N) conferring Imazapyr resistance and one silent point mutation to avoid DSB induction within the donor. For donor excision, the donor molecule is flanked by the respective spacer sequence (Figure 1a). The GT constructs, either containing *Lb*Cas12a or tt*Lb*Cas12a, were transformed as T‐DNAs into *Arabidopsis thaliana* Col‐0 plants via the floral dip method. After transformation, the T0 plants were grown either at 22°C or at 28°C until maturity and the harvested seeds subsequently sown out on Kanamycin selection medium. The resistant T1 primary transformants were grown until maturity at either 22°C or 28°C, respectively. The harvested T2 seeds were sown out on Imazapyr medium to verify ipGT events. At least 90 lines were tested per approach (Figure 1b). As expected, it turned out that raising the temperature from 22°C to 28°C is indeed enhancing GT efficiency. If we simply take the number of lines that produced resistant seedlings into account, tt*Lb*Cas12a outperforms the standard enzyme at 22°C by almost twofold. At 28°C, still one third more lines with GT events were detected for tt*Lb*Cas12a. The effect is even more pronounced if we take the number of GT events per line into account. For all lines, the individual GT frequencies were calculated by setting the total number of seeds into relation with the number of Imazapyr resistant seedlings. Mean GT frequencies were determined over all lines per approach. At 22°C, tt*Lb*Cas12a outperforms the native enzyme by 2.4‐fold and at 28°C still by 1.7‐fold. This is also obvious when we compare the targeting frequencies for the single lines individually for 28°C and for 22°C (Figure 1c and d). The difference in numbers and frequencies are especially impressive for the lower temperature but still clearly visible for the higher. Additionally, molecular analysis was performed to check the nature of GT events. DSB induced GT depends on the synthesis‐dependent strand annealing (SDSA) mechanism of HR (Huang and Puchta, 2019). In principle, besides perfect GT events, using HR at both junctions at the target locus also with a combination of HR and NHEJ, can lead to the restoration of the marker gene. Previously, we were able to demonstrate that in a large number of cases the marker gene is first restored by a homologous interaction with the target locus but then the vector integrates elsewhere in the genome by NHEJ. These ectopic targeting events can be discriminated from perfect GT events by PCR analysis of both donor‐genome junctions. We applied the same kind of analysis that we established in previous studies using the *ALS* gene as target (Wolter *et al*., 2018; Wolter and Puchta, 2019). All in all, over one hundred Imazapyr‐resistant plants, all representing independent GT events, were analysed. The respective result is shown in Figure 1e: the GT events induced by tt*Lb*Cas12a are in about half of the cases true GT events indicating that the modified enzyme, tt*Lb*Cas12a, is at least as efficient as the native one, which showed perfect GT events in about one third of the cases. Furthermore, for tt*Lb*Cas12a at 28°C molecular analysis indicated that 3% of the analysed T2 lines already comprised a homozygous GT event, which did not occur in any other approach in this study.

Recent results already demonstrated the beneficial effect of increased temperature on *Lb*Cas12a‐mediated GT in plants (van Vu *et al*., 2020). Here, we showed that the application of the recently developed tt*Lb*Cas12a (Schindele and Puchta, 2019) is not only an alternative way to boost GT for plants that cannot cope with high temperatures. GT can be elevated even further by its use in combination with a high‐temperature treatment. Yet, the potential of tt*Lb*Cas12a for GT might be even more promising as our data indicate. We were recently able to show that tt*Lb*Cas12a provides access to target sites that could hardly be edited at all before by *Lb*Cas12a even with a high‐temperature treatment (Schindele and Puchta, 2019). As we used a target locus that was accessible for native *Lb*Cas12a (Wolter and Puchta, 2019), the use of tt*Lb*Cas12a might increase GT efficiencies at those loci that are more difficult to access even much stronger.

## Conflict of Interest

The authors declare no conflict of interest.

## Author contributions

H.P. conceived the research. P.S., F.W., T‐K.H. and L.M. designed and L.M. executed the experiments. H.P., P.S. and L.M. wrote the article.
